# Genome Analysis of *Celeribacter* sp. PS-C1 Isolated from Sekinchan Beach in Selangor, Malaysia, Reveals Its β-Glucosidase and Licheninase Activities

**DOI:** 10.3390/microorganisms10020410

**Published:** 2022-02-10

**Authors:** Nurfatini Radzlin, Amira Suriaty Yaakop, Kian Mau Goh, Kok Jun Liew, Iffah Izzati Zakaria, Ummirul Mukminin Kahar

**Affiliations:** 1 Malaysia Genome and Vaccine Institute, National Institutes of Biotechnology Malaysia, Jalan Bangi, Kajang 43000, Selangor, Malaysia; nurfatiniradzlin@gmail.com (N.R.); iffahizzati@nibm.my (I.I.Z.); 2 Department of Biochemistry, Faculty of Biotechnology and Biomolecular Sciences, Universiti Putra Malaysia, Serdang 43400, Selangor, Malaysia; 3 School of Biological Sciences, Universiti Sains Malaysia, Minden 11800, Pulau Pinang, Malaysia; 4 Department of Biosciences, Faculty of Science, Universiti Teknologi Malaysia, Skudai 81310, Johor, Malaysia; gohkianmau@utm.my (K.M.G.); kokjunliew@gmail.com (K.J.L.)

**Keywords:** β-glucosidase, β-glucan, carbohydrate-active enzymes, *Celeribacter*, glycoside hydrolase, licheninase, lignocellulose biomass, marine bacteria, *Rhodobacteraceae*, starch

## Abstract

A halophilic marine bacterial strain, PS-C1, was isolated from Sekinchan beach in Selangor, Malaysia. The 16S rRNA gene sequence analysis indicated that strain PS-C1 was associated with the genus *Celeribacter*. To date, there have been no reports on enzymes from the genus *Celeribacter*. The present study reports on the cellular features of *Celeribacter* sp. PS-C1, its annotated genome sequence, and comparative genome analyses of *Celeribacter* glycoside hydrolase (GH) enzymes. The genome of strain PS-C1 has a size of 3.87 Mbp and a G+C content of 59.10%, and contains 3739 protein-coding genes. Detailed analysis using the Carbohydrate-Active enZYmes (CAZy) database revealed that *Celeribacter* genomes harboured at least 12 putative genes encoding industrially important GHs that are grouped as cellulases, β-glucanases, hemicellulases, and starch-degrading enzymes. Herein, the potential applications of these enzymes are discussed. Furthermore, the activities of two types of GHs (β-glucosidase and licheninase) in strain PS-C1 were demonstrated. These findings suggest that strain PS-C1 could be a reservoir of novel GH enzymes for lignocellulosic biomass degradation.

## 1. Introduction

Marine environments are home to complex and diverse microorganisms that are yet to be discovered through appropriate microbial investigations. Currently, marine-derived halophilic bacteria are being explored to harness their novel enzymes and bioactive compounds as substitutes for many industrial applications [1,2,3,4].

The *Rhodobacteraceae* family is one of the bacterial lineages in marine ecosystems [5], and currently consists of 191 genera that are archived in the List of Prokaryotic names with Standing in Nomenclature (LPSN) database (https://www.bacterio.net; accessed 1 January 2022). Unlike the major industrial microbes of the *Bacillaceae* family [6,7,8], reports on the potential applications of members of the family *Rhodobacteraceae* are relatively scarce [9]. *Celeribacter* is one of the least studied genera in *Rhodobacteraceae*, with no reports available on its industrial-related enzymes. Earlier studies using whole cells suggested that *Celeribacter* spp. could potentially be used in bioremediation processes of heavy metals [10], organic compounds [11,12,13,14], volatile substances [15], and hydrocarbons [16,17,18]. Members of this genus are halophiles and Gram-negative, rod-shaped bacteria that thrive in various marine habitats (e.g., seawater, sea sediments, and mangrove soil) [19,20,21]. To date, 10 types of *Celeribacter* species have been described and listed in the LPSN database (accessed 1 January 2022). These include: *Celeribacter neptunius* DSM26471^T^, *Celeribacter baekdonensis* DSM27375^T^, *Celeribacter halophilus* ZXM137^T^, *Celeribacter indicus* P73^T^*, Celeribacter marinus* IMCC12053^T^, *Celeribacter naphthalenivorans* EMB201^T^, *Celeribacter manganoxidans* DY2-5^T^, *Celeribacter persicus* DSM100434^T^, *Celeribacter ethanolicus* NH195^T^, and *Celeribacter arenosi* KMM9024^T^ [19,20,21,22,23,24,25,26,27,28,29].

The genome data for almost all members of the *Rhodobacteraceae* family are available on the National Center for Biotechnology Information (NCBI) Genome database (accessed 1 January 2022), with most studies related to the genera *Roseobacter* (257 projects), *Staleya* (223 projects), and *Ruegeria* (167 projects). In contrast, genomic studies on *Celeribacter* spp. are limited, with only 15 projects (including this study) deposited in the repository. Among the *Celeribacter* genomes, *C. ethanolicus* TSPH2 [30], *C. indicus* P73^T^ [16,24], and *C. marinus* IMCC12053^T^ [25,31] genomes have been completely sequenced, whereas the remaining 12 are draft genomes [19,26,27,32]. Therefore, genomic studies on *Celeribacter* spp. are crucial for understanding their biochemical networks and to disclose their biotechnological application prospects.

Lignocellulosic biomass consisting of cellulose, hemicellulose, and lignin serves as a raw material for second-generation biofuel production [33]. The complete degradation of lignocellulose involves a combination of numerous enzymes that can be categorised into three types based on their substrate specificities: cellulase, hemicellulase, and ligninolytic enzymes [34]. Cellulases, including β-glucosidase (EC 3.2.1.21), endoglucanase (EC 3.2.1.4), and exoglucanase (EC 3.2.1.91), are a group of enzymes that target the typical plant cell wall composed of cellulose [4]. β-Glucanases, such as endo-1,3(4)-β-glucanase (EC 3.2.1.6) and licheninase (EC 3.2.1.73), are active on the β-glucan cell wall of cereals, fungi, and seaweed, and have been reported to improve cellulose degradation [35,36]. Based on the Carbohydrate-Active enZYmes (CAZymes) classification, cellulases and β-glucanases belong to the glycoside hydrolase (GH) families GH1, GH3, GH5, GH6, GH7, GH8, GH9, GH10, GH12, GH16, GH19, GH26, GH30, GH44, GH45, GH48, GH51, GH61, GH74, GH116, and GH124 [4,37]. In addition, hemicellulases can degrade hemicellulose components such as xylan, mannan, and arabinan [38]. Examples of hemicellulases include xylanase (EC 3.2.1.8), β-xylosidase (EC 3.2.1.37), β-mannosidase (EC 3.2.1.25), α-galactosidase (EC 3.2.1.22), and α-L-arabinofuranosidase (EC 3.2.1.55). In the CAZy database, hemicelluloses were classified in the GH families 2, 5, 6, 7, 8, 9, 10, 11, 12, 16, 26, 30, 31, 36, 43, 44, 45, 48, 51, 61, 74, 95, and 124 [37,39]. Ligninolytic enzymes (e.g., laccase, EC 1.10.3.2; lignin peroxidase, EC 1.11.1.7; and manganese peroxidase, EC 1.11.1.13) are involved in the breakdown of lignin and primarily belong to the CAZy auxiliary activities (AA) families AA1 to AA7 [37,40]. The ability of *Celeribacter* spp. to deconstruct lignocellulosic biomass is unknown; however, preliminary analyses using commercial microbiology test kits have shown that *Celeribacter* cells might exhibit β-glucosidase activities [21,22,27].

The Sekinchan beach, located in Selangor, Malaysia (3.5029° N, 101.0945° E), is an underexplored site for microbial and enzyme research. Our previous studies using samples from Sekinchan beach revealed two bacterial strains (denoted as *Roseovarius* sp. PS-C2 and *Cellulomonas* sp. PS-H5), both of which may have various biotechnological applications [41,42]. In the present study, we aimed to elucidate the characteristics of a newly isolated bacterium from Sekinchan beach, designated as strain PS-C1, which belongs to the genus *Celeribacter.* The genomic features of strain PS-C1 are described herein. Additionally, we provided a comparative analysis of the GHs in strain PS-C1 and all 14 available *Celeribacter* genomes. Subsequently, we assessed the ability of strain PS-C1 to produce two types of GHs, β-glucosidase (BglPS-C1) and licheninase (LicPS-C1). To the best of our knowledge, this is the first report of a comparative analysis of *Celeribacter* GHs, and the first report on GH enzymes from *Celeribacter* spp.

## 2. Materials and Methods

### 2.1. Reagents and Chemicals

Unless otherwise stated, the chemicals were of analytical and molecular grade, and were purchased from Merck KGaA (Darmstadt, Germany). Marine broth was purchased from Condalab (Torrejón de Ardoz, Madrid, Spain). Marine agar was prepared by solidifying marine broth with 1.5% (*w*/*v*) V-agar (Condalab). Ampicillin, penicillin G, and chloramphenicol were obtained from BioBasic (Amherst, NY, USA), Amresco (Solon, OH, USA), and Sigma-Aldrich (St. Louis, MO, USA), respectively. Kanamycin sulphate and tetracycline hydrochloride were purchased from Calbiochem (San Diego, CA, USA). High-grade (≥98% purity) cellobiose, cellotriose, *p*-nitrophenyl-β-D-glucopyranoside (*p*NPG), and β-glucan from barley were obtained from Megazyme (County Wicklow, Ireland, UK).

### 2.2. Sampling Site, Isolation, Taxonomy Identification, and Bacterial Characterisation

Wet sediment and mud samples (uppermost layer until a depth of 15 cm) were collected using a sterilised laboratory scoop at Sekinchan beach in Selangor, Malaysia, on 14 September 2020. At the sampling site, the collected samples were stored in sterile bottles that were closed immediately after sampling. The samples were stored at 25 °C, transferred to the laboratory, and stored at 4 °C until further use. The temperature and pH of the collected samples were measured using a laboratory thermometer and pH meter, respectively.

Strain PS-C1 was isolated from the samples using a previously described ex situ cultivation method [41,42]. Pure colonies of strain PS-C1 were obtained by streaking the cells on marine agar at 30 °C (pH 6.5) for 48 h. Subsequently, genomic DNA was extracted from strain PS-C1 using the Monarch Genomic DNA Purification Kit (New England BioLabs, Ipswich, MA, USA) following the manufacturer’s instructions. The 16S rRNA gene was amplified by PCR using the forward primer 27F (5′-AGAGTTTGATCCTGGCTCAG-3′) and reverse primer 1492R (5′-GGTTACCTTGTTACGACTT-3′) [43]. Gene sequencing was performed by Apical Scientific Sdn. Bhd. (Sri Kembangan, Selangor, Malaysia). Taxonomic identification was performed by comparing the strain PS-C1’s 16S rRNA gene sequence with the available sequences in the NCBI GenBank and EzBioCloud 16S databases [44]. A phylogenetic tree was constructed using the neighbour-joining method with 1000 bootstrap replicates with MEGA11 software [45].

Field emission scanning electron microscopy (FESEM) was used to determine the cell shape and size of the strain PS-C1. The cells were treated and sputtered with gold according to the method established by Yang et al. [46] prior to observation under high-resolution FEI Quanta 650 FEG FESEM (Thermo Fisher Scientific, Hillsboro, OR, USA) operating at 10 kV. Gram staining and endospore detection were performed using the methods described by Stankus et al. [47] and observed under a light microscope (OPTIKA Srl, Ponteranica, Italy). Catalase and oxidase activities and hydrolysis of Tween 20 and Tween 80 were performed and assessed according to the method established by Beveridge et al. [48].

The temperature range and optimum growth of strain PS-C1 were analysed by incubating the cells in marine broth at 10–70 °C with shaking at 200 rpm for up to 3 days. The optimal pH for growth was determined at 30 °C and was tested over a pH range of 5.5–11.0. The salt tolerance of strain PS-C1 was determined in marine broth supplemented with 2–10% (*w*/*v*) NaCl. Cell growth was determined by measuring absorbance at 600 nm using an a Ultrospec 2100 *pro* U*V*/*V*isible Spectrophotometer (Cytiva, Marlborough, MA, USA).

The anaerobic growth conditions of strain PS-C1 were tested on marine agar slants at 30 °C using a sterile anaerobic jar for 7 days [49]. Susceptibility to antibiotics was investigated on marine agar spread with strain PS-C1 cells at 30 °C using the disc diffusion method [50] containing ampicillin (50 µg/mL), penicillin G (50 µg/mL), kanamycin sulphate (100 µg/mL), tetracycline hydrochloride (100 µg/mL), and chloramphenicol (100 µg/mL).

The motility test of strain PS-C1 was performed using the Analytical Profile Index (API) M Medium kit (bioMérieux, Marcy-l’Étoile, France). API 20NE and API 20E test strips (bioMérieux) were used to determine the basic biochemical characteristics of strain PS-C1. Carbohydrate utilisation and selective enzyme activity of strain PS-C1 were assessed using the API 50CH and API ZYM test strips (bioMérieux), respectively. All tests using API kits were performed at 30 °C according to the manufacturer’s protocol. Unless otherwise specified, all the aforementioned bacterial physiochemical and chemotaxonomic characterisations were performed in triplicate.

### 2.3. Genome Sequencing, Assembly, and Annotation

Strain PS-C1 was grown on marine agar (pH 6.5) at 30 °C for 24 h. Subsequently, strain PS-C1 genomic DNA was extracted from a single colony of cells using the standard protocol of the Monarch Genomic DNA Purification Kit (New England BioLabs). A paired-end library was prepared using the NEBNext Ultra DNA Library Prep Kit for Illumina (New England BioLabs), following the manufacturer’s instructions. Sequencing was performed using the NovaSeq 6000 system with 150 bp paired-end reads (Illumina, San Diego, CA, USA). Sequence adaptors and low-quality reads were filtered using Trimmomatic v.0.40 [51]. De novo genome assembly was performed using SOAPdenovo v.2.0.4 [52]. The assembled genome was analysed and annotated using the NCBI Prokaryotic Genome Annotation Pipeline (PGAP) v.5.20 [53]. Next, the protein-coding genes were clustered into functional groups using evolutionary genealogy of genes: Non-supervised Orthologous Groups (eggNOG) v.5.0 [54]. Metabolic pathways were predicted using BlastKOALA v.2.2 [55] based on the Kyoto Encyclopedia of Genes and Genomes (KEGG) database. Genome comparison between strain PS-C1 and all 14 available genomes of *Celeribacter* spp. in the NCBI Genome database (available as of 1 January 2022) was performed using digital DNA-DNA hybridisation (dDDH) in the Genome-to-Genome Distance Calculator (GGDC) v.2.1 [56] and the average nucleotide identity (ANI) function in the EzBioCloud server [57]. Default parameters were used for all software tools unless otherwise specified.

### 2.4. Analysis of CAZymes and Mining of GHs

The putative genes encoding CAZymes present in the genome of strain PS-C1 and all 14 available genomes of *Celeribacter* spp. were mined using the dbCAN2 meta server [58]. The InterProScan v.5.53-87.0 [59] and PSORTb v.3.0.3 [60] online servers were used to predict the protein domains and localisation of the annotated GHs, respectively. Unless otherwise specified, default parameters were used for all the software tools.

### 2.5. Expression and Determination of BglPS-C1 and LicPS-C1 Activities

Strain PS-C1 was grown on marine agar (pH 6.5) at 30 °C for 24 h. A single colony of strain PS-C1 was inoculated into 50 mL of marine broth in a 250 mL flask and shaken at 200 rpm at 30 °C for 24 h. To induce the expression of both BglPS-C1 and LicPS-C1 enzymes, a 20 mL inoculum (equivalent to 10% *v*/*v*) was aseptically transferred into 200 mL of marine broth supplemented with 1.0% (*w*/*v*) cellobiose in a 1 L flask. All the flasks were incubated at 30 °C with shaking at 200 rpm. At periodic time intervals, 5 mL of culture medium was sampled for up to 30 h. The absorbance was recorded at 600 nm using the Ultrospec 2100 *pro* U*V*/*V*isible Spectrophotometer (Cytiva), and the cells and cell-free supernatant were separated by centrifugation at 5000× *g* for 15 min at 4 °C. The cells and cell-free supernatant were stored at −80 °C and −20 °C, respectively, until further use. To obtain BglPS-C1 (intracellular enzyme), the cell pellets were lysed using the B-PER^TM^ Bacterial Protein Extraction Reagent kit (Thermo Fisher Scientific, Rockford, IL, USA), according to the manufacturer’s instructions. The cell-free lysate was dialysed against 100 mM sodium phosphate buffer (pH 6.5) for 18 h at 4 °C using SnakeSkin dialysis tubing with a 10 kDa molecular weight cut-off (Thermo Fisher Scientific). Subsequently, β-glucosidase activity was determined according to the method described by Chan et al. [61]. A reaction mixture containing 200 μL crude BglPS-C1 and 800 μL 10 mM *p*NPG in 100 mM sodium phosphate buffer (pH 6.5) was incubated at 50 °C for 15 min. The reaction was stopped by the addition of 1 mL 1 M Na_2_CO_3_. Subsequently, the release of *p*-nitrophenol was measured at 405 nm using the Ultrospec 2100 *pro* U*V*/*V*isible Spectrophotometer (Cytiva) at 405 nm. As a control, a reaction mixture without the enzyme was incubated and measured under the same conditions. *p-*Nitrophenol was used as the assay standard. One unit (U) of β-glucosidase activity was defined as the amount of enzyme that produced 1 µmol of *p*-nitrophenol per min per mL under the assay conditions. The enzyme activities were measured at least in triplicate, unless otherwise specified. To determine LicPS-C1 activity (extracellular enzyme) in the bacterial strain, the cell-free supernatant was allowed to react with *p*NPG (containing only β-1,4 glycosidic bonds) and β-glucan (containing both β-1,4 and β-1,3 glycosidic bonds). LicPS-C1 activity towards *p*NPG was determined in a similar manner as that for BglPS-C1. One unit (U) of licheninase activity was defined as the amount of enzyme that produced 1 µmol of *p*-nitrophenol per min per mL at 50 °C. LicPS-C1 activity towards natural substrates (β-glucan) was determined using the 3,5-dinitrosalicylic acid (DNS) method [62]. 500 μL each of crude LicPS-C1 and 1% (*w*/*v*) β-glucan dissolved in 100 mM sodium phosphate buffer (pH 6.5) were mixed and incubated at 50 °C for 30 min. DNS (1 mL) was then added to the mixture, followed by boiling for 5 min. Subsequently, the absorbance was measured at 540 nm using the Ultrospec 2100 *pro* U*V*/*V*isible Spectrophotometer (Cytiva). As a control, the unreacted mixture was incubated and analysed under the same conditions. Glucose was used as the assay standard. One unit (U) of licheninase activity was defined as the amount of enzyme that generated 1 µmol of reducing sugar per min per mL at 50 °C. All enzyme assays were performed in triplicate, unless otherwise specified. The reaction products of LicPS-C1 on β-glucan were analysed using ultra-high-performance liquid chromatography with an evaporative light-scattering detector (UHPLC-ELSD). The enzymatic reaction mixture was prepared by incubating crude LicPS-C1 with 1% (*w*/*v*) β-glucan in 100 mM sodium phosphate buffer (pH 6.5) at 50 °C for 48 h. At certain time intervals, the sample was withdrawn and the enzymatic reaction was stopped by boiling for 10 min. The insoluble particles were filtered through a 0.22 µm nylon-membrane syringe filter (Millex-GN, Merck Millipore, Darmstadt, Germany). A Shimadzu Nexera X2 UHPLC system with Shimadzu Nexera X2 ELSD (Shimadzu, Kyoto, Japan) and Rezex RSO-Oligosaccharide Ag^+^ column (10 × 200 mm; Phenomenex, Torrance, CA, USA) were used for the UHPLC-ELSD analysis. The column temperature was maintained at 80 °C. The ELSD nebuliser and evaporator temperatures were maintained at 30 °C, and standard N_2_ gas flow was maintained at 1.6 standard litres per min. Water (100% *v*/*v*) was used as the mobile phase at a flow rate of 0.2 mL/min. Glucose (Dp_1_), cellobiose (Dp_2_), and cellotriose (Dp_3_) were used as standards for the analyses. Unreacted substrate was used as the control.

All results of the enzymatic assays and UHPLC-ELSD analyses were statistically analysed using SYSTAT 12 software (Systat Software, San Jose, CA, USA). A Student’s *t-*test yielded a probability value (*p*-value) of less than 0.05, confirming that the data were adequate to test all hypotheses.

## 3. Results and Discussion

### 3.1. Sampling Site, Isolation, Taxonomy Identification, and Bacterial Characterisation

Sekinchan beach, located in Selangor, Malaysia, is a hot spot for tourism. The beach landscape is sandy with tiny grains of rocks, and some parts of the beach are muddy and rich in mangrove trees. The temperature and pH of the collected samples were 30–32 °C and pH 5.5–6.5, respectively.

**Figure 1:**
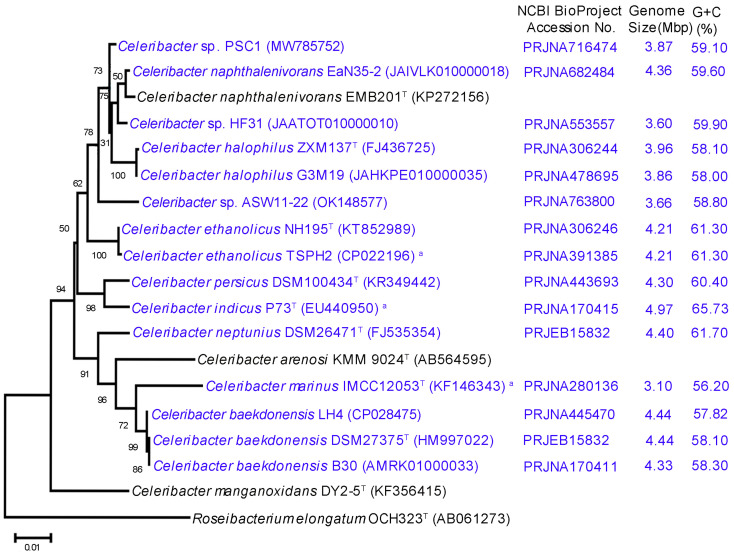
Phylogenetic tree based on 16S rRNA gene sequences showing the relationship between strain PS-C1 and representatives of *Celeribacter* spp. [19,20,21,22,23,24,25,26,27,28,29,30,31,32]. The 16S rRNA NCBI GenBank accession number for each strain is shown in brackets. The 16S rRNA sequences were aligned using ClustalW, and the tree was constructed using a neighbour-joining method with 1000 bootstrap replicates embedded in the MEGA11 software package [45]. The sequenced genomes and their respective information are indicated in blue. The scale bar represents 0.01 nucleotide substitution per site. *Roseibacterium elongatum* OCH323^T^ was used as an out-group. ^T^ Type strain; ^a^ complete genome.

In this study, we isolated a halophilic and cellulolytic bacterium designated as strain PS-C1 using an ex situ cultivation technique [41,42] from samples collected at Sekinchan beach. Strain PS-C1 is Gram-negative, and has light pink pigmentation. The 16S rRNA gene sequence (1453 bp) of strain PS-C1 was searched against the NCBI GenBank and EzBioCloud 16S databases [44]. Our analyses showed that strain PS-C1 was closely related to *C. naphthalenivorans* EMB201^T^ [21], with NCBI Blastn and EzBioCloud pairwise alignment values of 99.38% and 98.92%, respectively. The constructed 16S rRNA-based phylogenetic tree demonstrated that strain PS-C1 was clustered together (average sequence identity of 99%) with *C. naphthalenivorans* EaN35-2 (NCBI GenBank accession number JAIVLK010000018), *C. naphthalenivorans* EMB201^T^ [21], *Celeribacter* sp. HF31 (JAATOT010000010), *C. halophilus* ZXM137^T^ [22,24], and *C. halophilus* G3M19 (JAHKPE010000035) (Figure 1 and Table 1). In addition, strain PS-C1 had low identity (<98.36%) with the remaining species within the genus *Celeribacter* (Table 1 and Figure 1).

**Figure 2:**
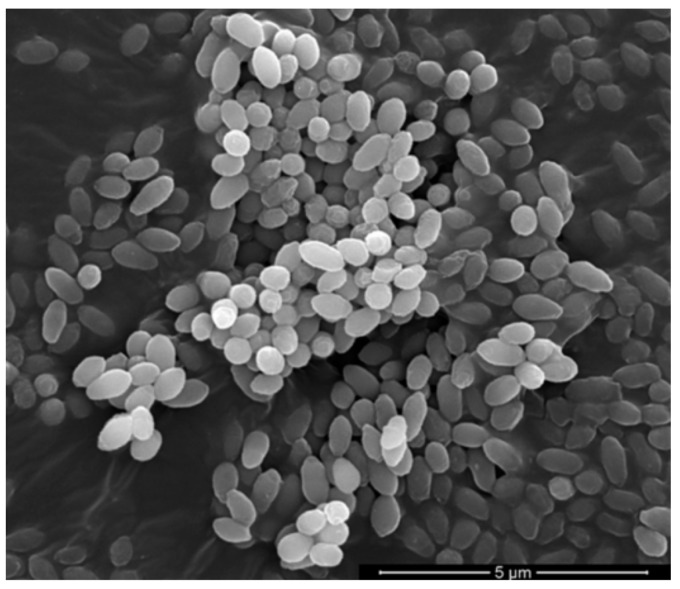
A field emission scanning electron micrograph of strain PS-C1 at 24,000× magnification. Scale bar: 5 μm.

FESEM observation at 24,000× magnification showed that the PS-C1 cells were rod-shaped, 0.18–1.2 µm wide, and 1.2–3.4 µm long (Figure 2 and Table 1).

*Celeribacter* spp. cells are singular and not filamentous or chain-shaped [19,23,24]. Similar to members of the genus *Celeribacter*, strain PS-C1 was positive in the catalase and oxidase tests, but negative in the motility test (Table 1). Except for *C. neptunius* DSM26471^T^, the strain tested negative in the oxidase test [19]. Strain PS-C1 was also found to be a non-spore-forming bacterium, and was unable to hydrolyse Tween 20 and Tween 80. These observations were concurrent with other *Celeribacter* spp., except for *C. indicus* P73^T^, *C. manganoxidans* DY2-5^T^, *C. naphthalenivorans* EMB201^T^*,* and *C. neptunius* DSM26471^T^, which could hydrolyse Tween 20 and Tween 80 [19,20,21,24].

The growth of strain PS-C1 was observed at temperatures of 30–40 °C and pH 5.5–10.0. The optimal growth conditions were determined to be 30 °C and pH 6.5. These results indicated that strain PS-C1 is a mesophilic and mildly acidophilic bacterium. *Celeribacter* spp. are known to be mesophiles with optimal growth temperatures of 25–30 °C, except for *C. ethanolicus* NH195^T^, which grows optimally at 37 °C (Table 1). Furthermore, most *Celeribacter* spp. are moderate alkaliphiles and grow optimally at neutral pH (Table 1). However, the optimal pH for growth of *C. baekdonensis* DSM27375^T^ and *C. persicus* DSM100434^T^ is pH 5.0 and pH 6.0, respectively, under acidic conditions [23,26]. Salt tolerance studies indicated that strain PS-C1 growth occurred at NaCl concentrations of 1–8% (*w*/*v*), with optimal growth at 2–7% (*w*/*v*) NaCl. The results showed that strain PS-C1 was a halophile, similar to all members of the genus *Celeribacter* (Table 1). Strain PS-C1 was found to be a facultative anaerobic bacterium (that could grow with and without oxygen). Among the members of the genus *Celeribacter*, only *C. neptunius* DSM26471^T^ [19] and *C. indicus* P73^T^ [24] are facultative anaerobes, while other *Celeribacter* spp. are strict aerobes (Table 1).

Antibiotic susceptibility tests showed that strain PS-C1 was unable to grow in the presence of ampicillin (50 µg/mL), penicillin G (50 µg/mL), kanamycin sulphate (100 µg/mL), tetracycline hydrochloride (100 µg/mL), and chloramphenicol (100 µg/mL). *Celeribacter* spp. are non-antibiotic-resistant bacteria; therefore, the antibiotic susceptibility profile of strain PS-C1 matched with that of other members of the genus *Celeribacter* (Table 1).

Based on the data obtained from the API 20NE, API 20E, and API 50CH analyses, strain PS-C1 was able to utilise a wide range of chemicals and carbon sources, including nitrate, citrate, urea, D-glucose, L-arabinose, D-fucose, and arbutin (Table 1 and Table S1). 

Selective enzymatic reactions of strain PS-C1 were identified using API ZYM, and the activities of esterase (C4), leucine arylamidase, acid phosphatase, α-glucosidase, and β-glucosidase were detected (Table 1 and Table S1). Other members of the genus *Celeribacter* can also degrade/uptake various substances. For more information, readers may refer to the literature on *Celeribacter* type strains, as listed in Table 1. Collectively, the results of phylogenetic analysis and phenotypic and chemotaxonomic properties indicated that strain PS-C1 belongs to the genus *Celeribacter*.

### 3.2. Genome Sequencing, Assembly, and Annotation

The genome of strain PS-C1 was sequenced, and its genomic features are summarised in Table 2.

The sequencer generated 1,671,186,900 bases from 5,570,623 paired-end reads. The genome was assembled into 40 contigs and showed a coverage of 357-fold. The largest contig was 499,873 bp, with N_50_ and N_90_ values of 302,457 bp and 80,703 bp, respectively. The draft genome size of strain PS-C1 was determined to be 3,866,278 bp (3.87 Mbp), which is smaller than that of other members of *Celeribacter* spp., such as *C. neptunius* DSM26471^T^ (4.97 Mb), *C. indicus* P73^T^ (4.40 Mbp), *C. naphthalenivorans* EaN35-2 (4.36 Mbp), and *C. ethanolicus* NH195^T^ (4.21 Mbp), but larger than *C. marinus* IMCC12053^T^ (3.10 Mbp) (Table S2 and Figure 1). An in-depth analysis using the collective information of *Celeribacter* genomes (Table S2) indicated that *Celeribacter* has a 44% larger genome size than its closest genus in the family *Rhodobacteraceae*: *Nereida* (~2.87 Mbp) [63]. The G+C content of strain PS-C1 was 59.10%, which was slightly lower than that of *C. naphthalenivorans* EaN35-2 (59.60%) and *Celeribacter* sp. HF31 (59.90%). Several members of *Celeribacter* spp. exhibited higher G+C content than strain PS-C1, such as *C. indicus* P73^T^ (65.73%), *C. neptunius* DSM26471^T^ (61.70%), and *C. ethanolicus* NH195^T^ (61.30%) (Table S2). On average, the G+C content of the genus *Celeribacter* (~59.62%) was higher than that of the closest genus *Nereida* (~54%) [63].

We then determined the taxonomic affiliation of strain PS-C1 by comparing its genome with all the available genomes of *Celeribacter* spp. (Table 3). Strain PS-C1 exhibited 18.20–37.50% dDDH and 72.50–89.30% ANI values with members of *Celeribacter* spp. The closest relative of strain PS-C1 was *C. naphthalenivorans* EaN35-2 (dDDH, 37.50%; ANI, 89.30%). As the values for dDDH (<70%) [64] and ANI (<96%) [57] were below the corresponding thresholds, these results indicated that strain PS-C1 is a new species of *Celeribacter*.

Based on the NCBI PGAP annotation, the strain PS-C1 genome consisted of 3818 predicted genes, of which 3739 were protein-coding sequences, 54 were noncoding RNA genes (48 tRNAs, 3 ncRNAs, and 3 rRNAs), and 25 were pseudogenes (Table 2). Of these, 1069 protein-coding sequences (28.59% of total protein-coding sequences) were found to be exclusive to the *Celeribacter* spp. with at least 90% sequence identity (Table S3). In addition, 2112 protein-coding sequences (56.49%) were associated (average sequence identity of 89.70%) with their respective counterparts from other genera in the family *Rhodobacteraceae*. A small portion of the proteins in the strain PS-C1 genome (96 proteins, 2.57%) were related (~67.72% identity) to their homologues from various bacterial families such as *Rhizobiaceae*, *Ahrensiaceae*, *Cohaesibacteraceae*, and *Halomonadaceae.* Furthermore, the strain PS-C1’s genome was found to encode a total of 462 hypothetical proteins (12.35% of the protein-coding sequences), as they shared low sequence identities with proteins available in the databases, and these proteins are interesting targets for future research. Compared to the closest member to strain PS-C1, the *C. naphthalenivorans* EaN35-2 genome had a total of 4206 protein-coding sequences (12.48% more than strain PS-C1), 1710 of which were identical (~92.36%) to their homologues from *Celeribacter* spp. (Table S3).

The protein-encoding sequences of strain PS-C1 were functionally categorised according to Cluster of Orthologous Groups (COGs) analysis, as shown in Table 4. A total of 3702 (87.51%) protein-coding genes were functionally assigned to COGs in the genome of strain PS-C1. Compared to the analysed *Celeribacter* genomes (Table S4), all strains had at least 77.67% of their total protein-encoding genes annotated with COG functions. In terms of COG assignment profiles, strain PS-C1 exhibited patterns similar to those of all the *Celeribacter* genomes. These genes were divided into four major functional groups: metabolism (30.27–46.58%), cellular processes and signalling (12.19–18.30%), information storage and processing (12.41–20.09%), and poorly characterised (15.22–18.99%) (Table S4).

An in-depth comparison between the genomes of PS-C1 and its closest relative *C. naphthalenivorans* EaN35-2 showed a major difference in the number of predicted genes related to the ‘(E)-amino acid transport and metabolism’ classification, with strain PS-C1 representing 324 genes (8.67%) in the genome, which is remarkably higher than *C. naphthalenivorans* EaN35-2 (three genes, 0.07%). In contrast, the genome of *C. naphthalenivorans* EaN35-2 (145 genes, 3.45%) had more genes assigned under the category ‘(L)-replication, recombination, and repair’ compared to that of strain PS-C1 (two genes, 0.05%) (Table S4). In terms of COG class for ‘(G)-carbohydrate transport and metabolism,’ a comparable amount of protein-encoding genes were grouped under this category for genomes of strain PS-C1 (219 genes, 5.86%), as well as *C. naphthalenivorans* EaN35-2 (211 genes, 5.02%). Some of these proteins (β-glucosidase, licheninase, and α-glucosidase) are known to be involved in the degradation of cellulose and starch [35,36,65,66].

Further inspection using KEGG metabolic pathway analysis indicated that the starch and sucrose metabolism pathways of strain PS-C1 and all the analysed *Celeribacter* genomes were relatively similar (Figure S1). According to this analysis, all strains encoded β-glucosidase (EC 3.2.1.2), which is necessary for the degradation of cellulose to glucose. Moreover, there were 15 enzymes present in all *Celeribacter* genomes predicted to be involved in the hydrolysis of starch to maltodextrin, maltose, and glucose; degradation of glycogen to glucose; and conversion of sucrose to D-fructose (Figure S1). In addition, the genomes of *C. baekdonensis* B30 and *C. indicus* P73^T^ harbour two additional enzymes. The genome of *C. baekdonensis* B30 was predicted to contain oligo-1,6-glucosidase (EC 3.2.1.10) and β-fructofuranosidase (EC 3.2.1.26), which are involved in the degradation of dextrin/isomaltose to glucose and conversion of sucrose-6-phosphate to glucose-6-phosphate, respectively. In contrast, the *C. indicus* P73^T^ genome contained alpha-trehalose-phosphate synthase (EC 2.4.1.15) and trehalose-phosphatase (EC 3.1.3.12) for the conversion of uridine diphosphate glucose to trehalose.

### 3.3. Analysis of CAZymes and Mining of GHs

**Figure 3:**
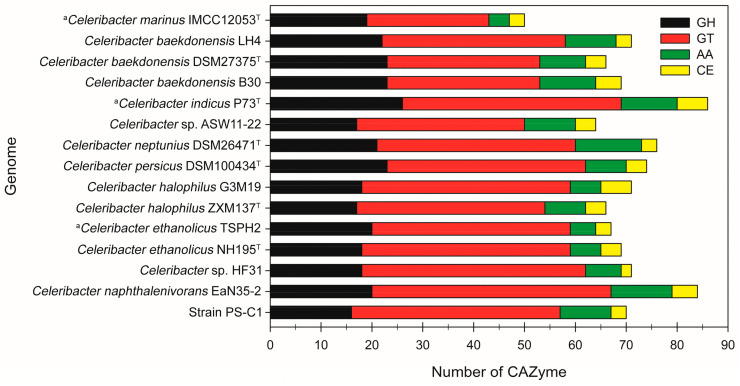
Total number of CAZymes present in strain PS-C1 and 14 other known genomes of *Celeribacter* spp. GH: glycoside hydrolase; GT: glycoside transferase; AA: auxiliary activity; CE: carbohydrate esterase; ^a^ complete genome; ^T^ type strain.

The dbCAN2 CAZy server was used to identify, predict, and compile the CAZyme-encoded genes in strain PS-C1 and 14 other analysed genomes of *Celeribacter* spp. An overview of the abundance and distribution of CAZymes in each member of the genus *Celeribacter* is shown in Figure 3.

The *Celeribacter* spp. encode a total of 50–86 different CAZymes. In terms of CAZyme classification, GHs and glycoside transferases (GTs) were the most dominant groups (average genes of 54.65% GH; ~29.17% GT) in all the genomes of *Celeribacter* spp. Moreover, small amounts of auxiliary activities (AAs) and carbohydrate esterases (CEs) were detected in *Celeribacter* spp. (~10.47% AAs; ~5.72% CE), whereas none of the *Celeribacter* spp. possessed encoded proteins assigned to polysaccharide lyases (Figure 3). Taken individually, the strain PS-C1 genome encoded a total of 70 CAZymes (including 16 GHs, 41 GTs, 10 AAs, and 3 CEs), whereas its closest relative *C. naphthalenivorans*EaN35-2 harboured a total of 84 CAZymes (20 GHs, 47 GTs, 12 AAs, and 5 CEs). The presence of various CAZymes in the genomes of *Celeribacter* spp. suggested that these proteins are likely responsible for the degradation of polysaccharides (i.e., cellulose and starch), and this hypothesis was in agreement with the carbon utilisation profiles of *Celeribacter* spp. (Table 1).

Currently, none of the CAZymes (particularly GHs) from *Celeribacter* spp. have been biochemically characterised. We analysed and compared the GHs in strain PS-C1 and all the 14 available genomes of *Celeribacter*. We hope that the analysis provided herein will pave the way for industrial applications of GH enzymes from *Celeribacter* spp. Table 5 lists the industrially relevant GH enzymes in *Celeribacter* genomes. The GHs shared among the analysed *Celeribacter* genomes could be divided into four categories according to the predicted carbohydrate-hydrolysing functions: (i) cellulose-degrading enzyme (one GH); (ii) β-glucan-degrading enzymes (three GHs); (iii) hemicellulose-degrading enzymes (five GHs); and (iv) starch-degrading enzymes (three GHs) (Table 5).

The GHs from *Celeribacter* spp. belonged to GH families 1, 2, 8, 13, 16, 26, 36, 43, and 51. Interestingly, all GHs shared low sequence identities (38.80–55.67%) with their closest orthologues from other genera available in the NCBI database (Table 5), which clearly indicated the novelty of *Celeribacter* GH enzymes.

For cellulose-degrading enzymes (also known as cellulase), the genes encoding GH1 β-glucosidase were consistently found in all *Celeribacter* genomes, with an average sequence identity of 76.13% (Table 5). From an industrial point of view, β-glucosidase has enormous potential for use in biofuel and food production. For example, β-glucosidase is the key enzyme that converts cello-oligosaccharides and cellobiose to glucose, which is used as feedstock for bioethanol production [4]. In the food industry, β-glucosidase has been applied as a flavour catalyst to remove aromatic compounds that impart an unpleasant taste to fruit juice, tea, and soy-based products [66]. Moreover, β-glucosidase may be used in the baking process to reduce dough viscosity, thus improving bread texture and quality [67]. In addition, it was found that all *Celeribacter* genomes encoded two types of β-glucan-degrading enzymes (β-glucanases) that are important for the breakdown of cellulose, curdlan, laminarin, and lichenin. Genes encoding GH16 licheninase and GH26 endo-β-1,3-1,4-glucanase were present in five distinct *Celeribacter* genomes (Table 5). Licheninase-encoding genes were detected in the genomes of strain PS-C1, *C. indicus* P73^T^, *C. halophilus* G3M19, *C. naphthalenivorans* EaN35-2, and *Celeribacter* sp. HF31 (average sequence identity of 64.11%). In contrast, the genes encoding endo-β-1,3–1,4-glucanase were present in the genomes of *C. ethanolicus* TSPH2, *C. baekdonensis* LH4, *C. neptunius* DSM26471^T^, *C. persicus* DSM100434^T^, and *C. ethanolicus* NH195^T^ (~88.57% identity). However, the genome of *Celeribacter* sp. HF31 exclusively harboured the GH8 endo-1,3(4)-β-glucanase gene (Table 5). We anticipated that Celeribacter spp. would require at least one type of GH enzyme that can degrade both the β-1,4 and β-1,3 glycosidic bonds of cellulose or β-glucan biomass as a cell adaptation strategy to acquire carbon sources for growth in their natural habitats (Figure S1). In biofuel production, licheninase, endo-β-1,3-1,4-glucanase, and endo-1,3(4)-β-glucanase have been used to depolymerise cellulose components, such as β-glucan [34,35,36].

In terms of hemicellulose-degrading enzymes (hemicellulases), a variety of annotated GHs was detected in the genomes of *Celeribacter* spp., which includes xylanases (GH26 β-1,3-xylanase and GH43 β-xylosidase), mannanases (GH2 β-mannosidase and GH36 α-galactosidase), and arabinofuranosidase (GH51 α-L-arabinofuranosidase) (Table 5). All hemicellulases shared 66.75–94.49% identity with their counterpart proteins within *Celeribacter* spp. However, the protein-encoding genes of β-xylosidase and α-L-arabinofuranosidase were exclusive to *C. marinus* IMCC12053^T^ and *C. naphthalenivorans* EaN35-2, respectively. In general, hemicellulases are used to disintegrate the major parts of hemicellulose, such as xylan, mannan, and arabinan to release xylose, mannose, and arabinose, respectively [7,33,38]. These sugars have been used as materials for biofuel production, bread formulation, and prebiotic-based products [4,67].

Three types of GH13 starch-degrading enzymes, α-amylase (EC 3.2.1.1), oligo-1,6-glucosidase (EC 3.2.1.10), and α-glucosidase (EC 3.2.1.20), were detected in *Celeribacter* genomes (Table 5). Among the analysed genomes, only the *C. baekdonensis* B30 genome was predicted to have all the genes encoding the three aforementioned starch-degrading enzymes. We found that α-glucosidase was present in all *Celeribacter* genomes, with an average sequence identity of 35.48% compared to the reference protein from *C. indicus* P73^T^. In bioindustries, α-glucosidase is used to degrade the α-1,4 glycosidic bonds of starch linear oligomers (i.e., malto-oligosaccharides and maltose) to produce glucose [65]. Based on the results shown in Table 5, oligo-1,6-glucosidase was only detected in the genome of *C. baekdonensis* B30. This enzyme catalyses the hydrolysis of 𝛼-1,6 glycosidic bonds in amylopectin, 𝛼-limit dextrins, and isomalto-oligosaccharides [65,68]. In the industrial starch saccharification process, oligo-1,6-glucosidase is used as a substitute for debranching enzymes (i.e., type I pullulanase and isoamylase) to produce sugar syrups [68]. Moreover, three of the analysed genomes (*C. halophilus* ZXM137^T^, *C. halophilus* G3M19, and *C. baekdonensis* B30) encoded genes for α-amylases. Interestingly, the α-amylase from *C. baekdonensis* B30 is unique among the genomes of *Celeribacter*, as the protein shared only 24.15% sequence identity compared to its reference homologue from *C. halophilus* ZXM137^T^. α-Amylase is one of the oldest industrial enzymes and is used in various applications, including starch liquefaction and saccharification processes, food and beverage production, as an additive in textile detergents, paper processing, animal feed formulation, and bioethanol production [1,65,69]. Recently, α-amylase has also been applied as catalyst in the wastewater bioremediation, medicinal tablet formulation, and pharmaceutical biofilm inhibitory products [69].

### 3.4. Expression and Determination of BglPS-C1 and LicPS-C1 Activities

**Figure 4:**
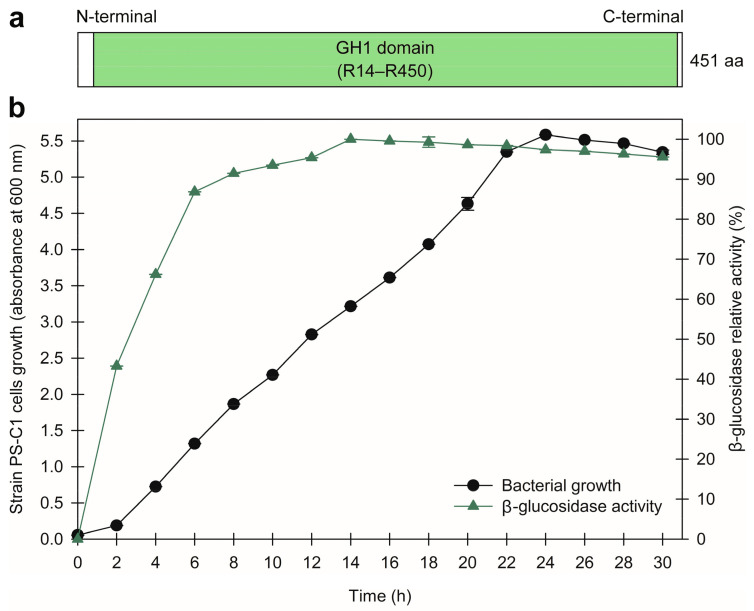
(**a**) Schematic representation of domain organisation for β-glucosidase in strain PS-C1 (BglPS-C1); aa: amino acids. (**b**) Time courses of growth of strain PS-C1 and BglPS-C1 production by cultivation in the marine broth supplemented with 1.0% (*w*/*v*) cellobiose. *p*-Nitrophenyl-β-D-glucopyranoside (*p*NPG) was used as the enzymatic substrate. The values shown represent the mean ± standard error of triplicate analyses.

We then expressed and elucidated the activities of two GHs (BglPS-C1 and LicPS-C1) in strain PS-C1. For BglPS-C1, the protein sequence (451 residues) consisted of a GH1 domain located at position R14–R450, as predicted using the InterProScan server (Figure 4a).

The GH1 domain is a catalytic region where the hydrolysis of β-1,4 glycosidic bonds occur [70]. In terms of enzyme localisation based on the PSORTb web server, BglPS-C1 was predicted to be an intracellular enzyme; thus, the cells were lysed prior to the enzymatic assays. As shown in Figure 4b, BglPS-C1 was constitutively produced throughout the 30 h time course, suggesting its important role in the conversion of cello-oligosaccharides in strain PS-C1. The maximum relative enzyme activity was determined after a 14 h incubation period, which was equivalent to the exponential phase of cell growth. Similar to other studies, the optimum β-glucosidase expression in *Fusobacterium* sp. K-60 and *Pseudomonas pickettii* were recorded during the exponential phase of growth [71,72].

**Figure 5:**
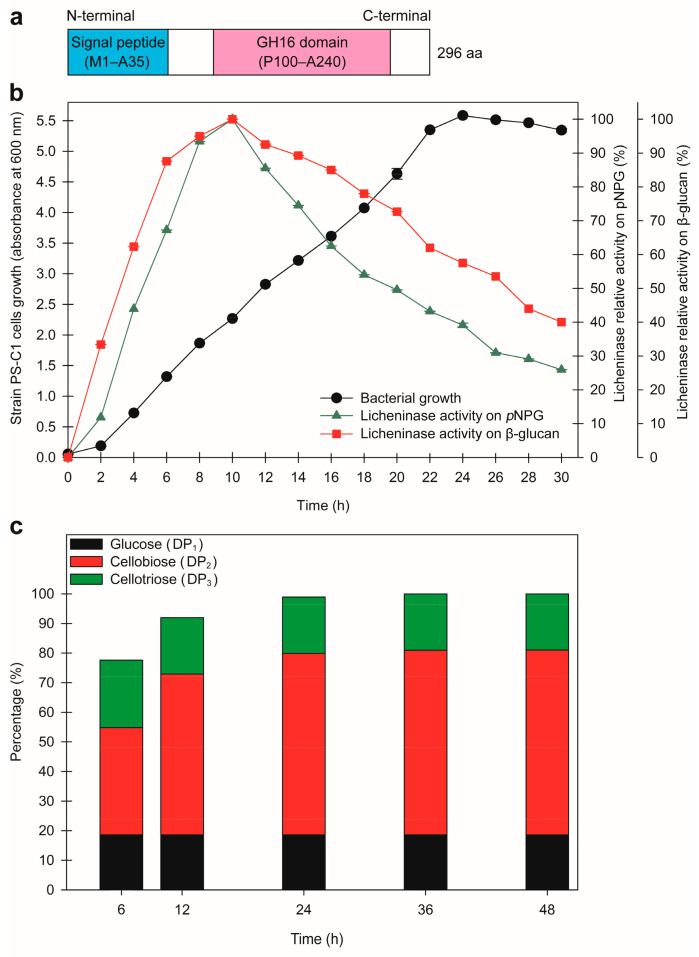
(**a**) Schematic representation of domain arrangements for the licheninase from strain PS-C1 (LicPS-C1); aa: amino acids. (**b**) Time courses of strain PS-C1 growth and LicPS-C1 production by cultivation in the marine broth supplemented with 1% (*w*/*v*) cellobiose. The LicPS-C1 activities were detected using *p*-nitrophenyl-β-D-glucopyranoside (*p*NPG) and β-glucan as the enzymatic substrates. The values shown represent the mean ± standard error of triplicate analyses. (**c**) Analysis of reaction products produced by LicPS-C1 acting on β-glucan at different time intervals using ultra-high-performance liquid chromatography with an evaporative light-scattering detector (UHPLC-ELSD). The amount of total sugars produced at each time point is shown relative to that at 48 h. Dp_1_: glucose; Dp_2_: cellobiose; Dp_3_: cellotriose.

In addition, the LicPS-C1 protein sequence (296 residues) is composed of a signal peptide (M1–A35) and a GH16 domain (P100–A240), which act as the extracellular secretion signal [73] and catalytic region [74], respectively (Figure 5a).

As LicPS-C1 was deduced to be an extracellular enzyme, the cell-free supernatant was used to measure the enzyme activities of *p*NPG (containing only β-1,4 glycosidic bonds) and β-glucan (containing both β-1,4 and β-1,3 glycosidic bonds). As shown in Figure 5b, LicPS-C1 could act on both *p*NPG and β-glucan, indicating that the enzyme was actively hydrolysing β-1,4 and β-1,3 glycosidic linkages. The LicPS-C1 expression pattern on both *p*NPG and β-glucan was as follows: the relative enzyme activities increased gradually from 0 to 8 h, reached their optimum at 10 h of incubation, and decreased within 12–30 h of incubation (Figure 5b). In separate studies, similar patterns were observed in the production of licheninase from *Bacillus subtilis* HL-25 and *Bacillus subtilis* GN156 at 72 h and 24 h of incubation, respectively [75,76]. The end-product (sugar) profile of LicPS-C1 on β-glucan over a 48 h time course was determined using UHPLC-ELSD analysis (Figure 5c). At the beginning of the time plot (6 h), the relative amount of total sugar produced was 77.62%. The total relative amount of sugar then increased to 98.94% at 24 h and reached its optimum (100%) after 36 h of incubation. In terms of types of sugars, glucose (Dp_1_), cellobiose (DP_2_), and cellotriose (DP_3_) were produced at various ratios throughout the analysis. The majority of sugars produced were cellobiose (36.22–62.41%) and cellotriose (19.02–22.79%). Glucose was released at a constant amount (~18.59%) over the 48 h reaction period. Altogether, the GHs of strain PS-C1 (i.e., BglPS-C1 and LicPS-C1) are interesting new enzymes for biomass degradation. Further studies on the gene cloning, purification, and functional biochemical characterisation of these enzymes may reveal their potential biotechnological applications.

## 4. Conclusions

In this report, we described phenotypic, chemotaxonomic, and phylogenetic analyses of strain PS-C1. These results collectively suggested that strain PS-C1 represents a new member of the genus *Celeribacter*. Additionally, we presented the genomic features of *Celeribacter* sp. PS-C1 and provided the first comprehensive analysis of the underexplored GHs within the genomes of *Celeribacter* spp. Furthermore, two GHs from *Celeribacter* sp. PS-C1 (β-glucosidase and licheninase) were expressed, and their activities were analysed. Based on genomic and experimental data, *Celeribacter* sp. PS-C1 is an attractive reservoir for novel GH enzymes that might be useful in biomass saccharification.

## Figures and Tables

**Table 1:** Comparison of morphology and biochemical characteristics between strain PS-C1 and type strains of the genus *Celeribacter.*

Characteristic	PS-C1 ^a^	EMB201^T^	ZXM137^T^	NH195^T^	DSM100434^T^	DSM26471^T^	P73^T^	DSM27375^T^	DY2-5^T^	KMM9024^T^	IMCC12053 ^T^
**Cell property**											
Cell size (width × length, µm)	0.18–1.2 × 1.2–3.4	0.8–1.2 × 1.2–3.4	0.3 × 0.8	0.5–1.0 × 1.0–2.0	0.4–0.5 × 0.8–0.9	0.4–0.9 × 0.8–1.8	0.6–0.7 × 1.2–1.3	0.6–1.0 × 1.0–3.0	0.5–0.8 × 1.2–2.1	0.6–0.8 × 2.5–4.5	0.6–0.7 × 1.4–2.3
Catalase	+	+	+	+	+	+	+	+	+	+	+
Oxidase	+	+	+	+	+	−	+	+	ND	+	+
Motility	−	−	−	−	ND	−	−	−	−	−	−
**Growth condition**											
Temperature range (optimum) (°C)	30–40 (30)	10–37 (30)	4–45 (28)	20–40 (37)	10–45 (28)	5–35 (25)	10–41 (28)	4–37 (30)	0–37 (28)	4–37 (25–30)	4–37 (30)
pH range (optimum)	5.5–10.0 (6.5)	5.0–9.5 (7.0–7.5)	6.0–9.0 (7.0)	5.0–9.0 (7.5)	5.0–9.0 (6.0)	7.5–8.0 (7.5–8.0)	2.0–12.0 (ND)	7.5–8.0 (5.0)	6.5–9.0 (7.0–7.5)	5.5–9.5 (7.9–8.0)	6.0–9.0 (8.0)
NaCl range (optimum) (% *w*/*v*)	1.0–8.0 (2.0–7.0)	1.0–7.0 (2.0–3.0)	0.5–11.0 (ND)	0.5–10.0 (1.0–3.0)	0–18.0 (3)	1.0–8.0 (ND)	0.5–12.0 (3.0)	0–13.0 (2.0)	11.0 (3.0–4.0)	1.0–7.0 (3.0–4.0)	0.5–5.0 (2.5–3.0)
Anaerobic growth	+	−	−	−	−	+	+	−	ND	−	−
**Antibiotic susceptibility**											
Ampicillin (50 µg/mL)	+	nd	+	+	nd	nd	+	+	nd	+	nd
Penicillin G (50 µg/mL)	+	nd	+	nd	nd	nd	nd	+	nd	+	nd
Tetracycline hydrochloride (100 µg/mL)	+	nd	+	+	nd	+	+	nd	nd	+	nd
Kanamycin sulphate (100 µg/mL)	+	nd	+	+	nd	nd	+	+	nd	+	+
Chloramphenicol (100 µg/mL)	+	nd	nd	+	nd	+	nd	+	nd	+	+
**API result (20NE, 20E, 50CH, and ZYM)**											
Nitrate reduction	+	−	−	−	+	+	−	−	−	+	−
Citrate utilization	+	−	+	−	−	−	−	+	−	−	nd
Indole production	−	−	−	−	nd	−	−	−	−	−	−
Urease degradation	+	−	+	+	+	+	+	+	+	−	−
D-lactose degradation	−	nd	−	−	−	+	nd	+	−	−	−
Lipase activity	−	−	+	−	+	−	+	−	−	−	−
α-glucosidase activity	+	+	+	+	+	+	−	+	nd	+	+
β-glucosidase activity	+	+	+	+	+	+	+	+	−	nd	+
**16S rRNA analysis against strain PS-C1**											
NCBI GenBank accession number	MW785752	KP272156	FJ436725	KT852989	KR349442	FJ535354	EU440950	HM997022	KF356415	AB564595	KF146343
NCBI Blastn (%)	100.00	99.38	99.04	98.36	97.01	96.94	96.72	96.37	95.10	94.46	94.27
EzBioCloud pairwise alignment (%) ^b^	100.00	98.92	99.06	98.41	96.83	96.89	96.61	96.54	95.38	94.59	94.66
**Reference**	This study	[21]	[22,24]	[27]	[26]	[19]	[24]	[23]	[20]	[28,29]	[25]

PS-C1: Strain PS-C1; EMB201^T^: *Celeribacter naphthalenivorans* EMB201^T^; ZXM137^T^: *Celeribacter halophilus* ZXM137^T^; NH195^T^: *Celeribacter ethanolicus* NH195^T^; DSM100434^T^: *Celeribacter persicus* DSM100434^T^; DSM26471^T^: *Celeribacter neptunius* DSM26471^T^; P73^T^: *Celeribacter indicus* P73^T^; DSM27375^T^: *Celeribacter baekdonensis* DSM27375^T^; DY2-5^T^: *Celeribacter manganoxidans* DY2-5^T^; KMM9024^T^: *Celeribacter arenosi* KMM9024^T^; IMCC12053^T^: *Celeribacter marinus* IMCC12053^T^. Characteristics are scored as: +, positive; −, negative; and, not determined. ^a^ Complete API result for strain PS-C1 is listed in Table S1; ^b^ pairwise 16S rRNA sequence alignment for taxonomy using EzBioCloud 16S database of strain PS-C1 against all type strains of *Celeribacter* spp.; ^T^ type strain.

**Table 2:** Genome attributes of strain PS-C1 according to Minimum Information about a Genome Sequence (MIGS) specification ^a^.

Attribute	Description
**Genome assembly statistics**	
Sequencing platform	Illumina NovaSeq
Assembly	SOAPdenovo v2.04
Finishing strategy	High-quality draft
Genome coverage	357 ×
Genome quality	No contamination
Relevance	Industrial
**Genome metrics**	
Genome size (bp)	3,866,278
G+C content (%)	59.10%
Number of contigs	40
Longest contig length (bp)	499,873
N_50_ value	302,457
N_90_ value	80,703
L_50_ value	5
Total genes	3818
Pseudogenes	25
Noncoding RNA genes	54
tRNA	48
ncRNA	3
5S rRNA	1
16S rRNA	1
23S rRNA	1
Protein-coding sequences	3739
With Pfam	2657
With signal peptide	496
With COGs	3702
Connected to KEGG pathways	2218
Putative hypothetical proteins	462
**NCBI accession number**	
GenBank	JAHXRW000000000
BioProject	PRJNA716474
BioSample	SAMN18354561
Locus Tag	J5Y17
GenBank	JAHXRW000000000

^a^ The MIGS information for all *Celeribacter* genomes is listed in Table S2.

**Table 3:** Genomic comparison of strain PS-C1 against all available genomes of *Celeribacter* spp. using Genome-to-Genome Distance Calculator (GGDC) and Average Nucleotide Identity (ANI).

	dDDH	Strain														
ANI		PS-C1	EaN35-2	HF31	NH195^T^	TSPH2	ZXM137^T^	G3M19	DSM100434^T^	DSM26471^T^	ASW11-22	P73^T^	B30	DSM27375^T^	LH4	IMCC12053^T^
**Strain**	**PS-C1**	100.00	**37.50**	34.60	23.80	23.70	22.60	22.60	22.50	22.40	21.10	19.80	19.70	19.50	19.40	18.20
	**EaN35-2**	**89.28**	100.00	41.10	24.90	24.40	23.10	23.00	22.80	23.00	20.90	20.30	19.90	19.70	19.80	18.90
	**HF31**	88.12	90.42	100.00	24.30	24.30	23.00	23.10	22.70	22.80	20.70	20.10	19.90	19.80	19.70	18.90
	**NH195^T^**	81.39	82.05	81.67	100.00	82.10	22.00	21.80	30.50	24.10	21.00	21.10	20.60	20.60	19.50	18.70
	**TSPH2**	81.10	81.73	81.60	98.07	100.00	21.90	21.90	29.80	23.00	20.70	22.00	19.70	19.90	19.40	19.00
	**ZXM137^T^**	80.31	80.37	80.55	86.07	85.62	100.00	61.30	21.70	22.00	20.20	19.90	19.40	19.60	19.70	19.00
	**G3M19**	80.18	80.43	80.44	79.25	79.23	79.04	100.00	21.60	22.00	20.20	19.80	19.40	19.40	19.40	18.70
	**DSM100434^T^**	79.95	80.33	80.45	79.06	78.97	78.98	95.22	100.00	23.50	20.30	21.20	20.70	21.00	19.20	18.30
	**DSM26471^T^**	79.73	80.36	80.04	80.81	80.18	80.44	78.68	78.68	100.00	20.80	21.10	21.50	21.20	19.70	18.90
	**ASW11-22**	77.70	77.64	77.11	77.44	77.29	77.14	76.23	76.37	77.11	100.00	19.30	18.70	18.40	18.60	18.20
	**P73^T^**	76.46	76.73	76.67	76.94	76.82	77.32	75.94	76.02	77.44	74.25	100.00	19.00	18.70	18.50	18.20
	**B30**	76.41	76.80	76.77	76.94	76.53	77.18	75.82	75.89	77.44	74.51	93.67	100.00	53.90	40.80	18.70
	**DSM27375^T^**	76.30	76.68	76.37	76.24	76.32	75.94	75.98	75.97	76.18	74.35	89.78	90.28	100.00	39.00	18.60
	**LH4**	76.23	76.91	76.99	77.90	78.92	78.27	75.99	75.93	77.48	75.43	74.53	74.90	74.60	100.00	72.10
	**IMCC12053^T^**	72.51	72.78	72.83	72.48	72.56	72.45	72.71	72.63	72.51	72.27	72.91	72.99	73.12	72.09	100.00

The dDDH and ANI values (%) shared between the genomes are shown at blue and red diagonals, respectively; Bold values: closest relative to strain PS-C1; PS-C1: strain PS-C1; EaN35-2: *Celeribacter naphthalenivorans* EaN35-2; HF31: *Celeribacter* sp. HF31; NH195^T^: *Celeribacter ethanolicus* NH195^T^ [27]; TSPH2: *Celeribacter ethanolicus* TSPH2 [30]; ZXM137^T^: *Celeribacter halophilus* ZXM137^T^; G3M19: *Celeribacter halophilus* G3M19; DSM100434^T^: *Celeribacter persicus* DSM100434^T^ [26]; DSM26471^T^: *Celeribacter neptunius* DSM26471^T^ [19]; ASW11-22: *Celeribacter* sp. ASW11-22; P73^T^: *Celeribacter indicus* P73^T^ [16,24]; B30: *Celeribacter baekdonensis* B30; DSM27375^T^*: Celeribacter baekdonensis* DSM27375^T^; LH4*: Celeribacter baekdonensis* LH4 [32]; IMCC12053^T^: *Celeribacter marinus* IMCC12053^T^ [25,31]; ^T^ type strain.

**Table 4:** Cluster of Orthologous Group (COG) functional assignments of strain PS-C1 ^a^.

COG Class	COG Functional Categories	Strain PS-C1	
		Gene Count	Percentage (%)
	**Metabolism**		
C	Energy production and conversion	254	6.79
E	Amino acid transport and metabolism	324	8.67
F	Nucleotide transport and metabolism	86	2.30
G	Carbohydrate transport and metabolism	219	5.86
H	Coenzyme transport and metabolism	126	3.37
I	Lipid transport and metabolism	125	3.34
P	Inorganic ion transport and metabolism	253	6.77
Q	Secondary metabolites, biosynthesis, transport, and catabolism	118	3.16
	**Cellular processes and signalling**		
D	Cell cycle control, cell division, and mitosis	37	0.99
M	Cell wall/membrane/envelope biogenesis	339	9.07
N	Cell motility	7	0.19
O	Post-translational modification, protein turnover, chaperone functions	117	3.13
T	Signal transduction mechanisms	119	3.18
U	Intracellular trafficking, secretion, and vesicular transport	65	1.74
	**Information storage and processing**		
J	Translation, ribosomal structure, and biogenesis	187	5.00
K	Transcription	240	6.42
L	Replication, recombination, and repair	2	0.05
V	Defense mechanisms	34	0.91
Z	Cytoskeleton	1	0.03
	**Poorly characterized**		
R	General function prediction only	0	0.00
S	Function unknown	619	16.56
	**Total**	3702	87.51

^a^ The complete COG profiles for all *Celeribacter* genomes are listed in Table S4.

**Table 5:** List of several glycoside hydrolases (GHs) identified in *Celeribacter* genomes and their identity to the other genera.

Category (CAZy GH Family)	Predicted function (EC Number)	Identity within *Celeribacter* Genome (%) ^a^															Identity to Other Genera (%)
		PS-C1	P73^T^	TSPH2	IMCC12053^T^	LH4	DSM27375^T^	DSM26471^T^	DSM100434^T^	NH195^T^	ZXM137^T^	G3M19	ASW11-22	EaN35-2	B30	HF31	
**Cellulose-Degrading Enzyme**																	
(GH1)	β-glucosidase (3.2.1.21)	73.53	100	73.76	75.28	75.57	75.79	71.69	74.89	73.76	75.11	75.11	71.72	74.66	76.47	74.66	*Acetivibrio* *thermocellus* (41.91)
**Β-glucan-Degrading Enzyme**																	
(GH8)	Endo-1,3(4)-β-glucanase (3.2.1.6)	−	−	−	−	−	−	−	−	−	−	−	−	−	−	100	*Bacillus circulans* (36.90)
(GH16)	Licheninase (3.2.1.73)	53.00	100	−	−	−	−	−	−	−	−	58.25	−	53.00	−	56.30	*Paenibacillus* *polymyxa* (42.71)
(GH26)	Endo-β-1,3-1,4- glucanase (3.2.1.73)	−	−	100	−	74.58	−	72.58	96.03	99.67	−	−	−	−	−	-	*Profundibacter amoris* (76.62)
**Hemicellulose-Degrading Enzyme**																	
(GH2)	β-mannosidase (3.2.1.25)	−	−	100	−	66.75	68.99	−	81.65	97.13	−	−	−	−	68.86	−	*Pacifibacter marinus* (55.67)
(GH26)	β-1,3-xylanase (3.2.1.32)	100	−	−	−	−	−	−	−	−	−	−	−	−	−	92.25	*Vibrio* sp. AX-4 (29.50)
(GH36)	α-galactosidase (3.2.1.22)	−	−	−	−	−	−	−	−	−	100	94.49	−	−	−	−	*Escherichia coli* (38.80)
(GH43)	β-xylosidase (3.2.1.37)	−	−	−	100	−	−	−	−	−	−	−	−	−	−	−	*Bacillus subtilis* (44.60)
(GH51)	α-L-arabinofuranosidase (3.2.1.55)	−	−	−	−	−	−	−	−	−	−	−	−	100	−	−	*Geobacillus stearothermophilus* (54.30)
**Starch-degrading enzyme**																	
(GH13)	α-amylase (3.2.1.1)	−	−	−	−	−	−	−	−	−	100	98.97	−	−	24.15	−	*Spirochaeta thermophila* (40.90)
(GH13)	Oligo-1,6-glucosidase (3.2.1.10)	−	−	−	−	−	−	−	−	−	−	−	−	−	100	−	*Bacillus halodurans* (51.70)
(GH13)	α-glucosidase (3.2.1.20)	30.97	100	31.06	30.77	29.87	30.42	30.76	31.67	31.23	31.19	31.36	31.03	31.29	30.00	30.62	*Bacillus halodurans* (48.60)

PS-C1: strain PS-C1; EaN35-2: *Celeribacter naphthalenivorans* EaN35-2; HF31: *Celeribacter* sp. HF31; NH195^T^: *Celeribacter ethanolicus* NH195^T^ [27]; TSPH2: *Celeribacter ethanolicus* TSPH2 [30]; ZXM137^T^: *Celeribacter halophilus* ZXM137^T^; G3M19: *Celeribacter halophilus* G3M19; DSM100434^T^: *Celeribacter persicus* DSM100434^T^ [26]; DSM26471^T^: Celeribacter neptunius DSM26471^T^ [19]; ASW11-22: *Celeribacter* sp. ASW11-22; P73^T^: *Celeribacter indicus* P73^T^ [16,24]; B30: *Celeribacter baekdonensis* B30; DSM27375^T^: *Celeribacter baekdonensis* DSM27375^T^; LH4: *Celeribacter baekdonensis* LH4 [32]; IMCC12053^T^: *Celeribacter marinus* IMCC12053^T^ [25,31]. Characteristics scored as: -, absence; ^T^ type strain. ^a^ The NCBI GenBank accession numbers for the respective GHs are listed in Table S5.

## Data Availability

The data for whole-genome shotgun sequencing of *Celeribacter* sp. PS-C1 are publicly available in NCBI GenBank under BioProject accession number PRJNA716474, BioSample accession number SAMN18354561, and GenBank accession number JAHXRW000000000. The version described in this paper is the first version, JAHXRW000000000.1. The raw sequencing reads were deposited in the NCBI Sequence Read Archive (SRA) under accession number SRR15464887. The 16S rRNA gene sequence of *Celeribacter* sp. PS-C1 was deposited in NCBI GenBank under accession number MW785752. The protein sequences of β-glucosidase and licheninase from *Celeribacter* sp. PS-C1 are publicly accessible under the NCBI GenBank accession numbers MBW6417521 and MBW6416931, respectively.

## Supplementary Materials

The following supporting information can be downloaded at: https://www.mdpi.com/article/10.3390/microorganisms10020410/s1, Figure S1: KEGG-based representation of starch and sucrose metabolism in strain PS-C1 and all analysed *Celeribacter* genomes. The β-glucosidase (EC 3.2.1.21) involved in cellulose metabolism is highlighted in red boxes; Table S1: Collective biochemical characterisation of strain PS-C1 using Analytical Profile Index (API) analyses; Table S2: Genome attributes of *Celeribacter* spp. according to Minimum Information about a Genome Sequence (MIGS) specifications; Table S3: The closest identities of proteins in the genomes of strain PS-C1 and *Celeribacter naphthalenivorans* EaN35-2; Table S4: Cluster of Orthologous Groups (COGs) functional assignments of protein-coding genes in *Celeribacter* genomes; Table S5: List of NCBI GenBank/RefSeq accession numbers for glycoside hydrolases (GHs) in *Celeribacter* genomes.
