# Supplementary material for: Genome Analysis of Celeribacter sp. PS-C1 Isolated from Sekinchan Beach in Selangor, Malaysia, Reveals Its β-Glucosidase and Licheninase Activities
Source: Microorganisms. 2022 Feb 10;10(2):410. doi: 10.3390/microorganisms10020410 (PMC8874975; doi:10.3390/microorganisms10020410)
Supplement: Supplementary file 1 [file microorganisms-10-00410-s001.zip › Table S1.pdf]

Table S1. Collective biochemical characterisation of strain PS-C1 using Analytical Profile Index (API) analyses

**API ZYM (enzyme reaction)**		**API 20E (substrate/chemical utilization)**		**API 20NE (substrate/chemical utilization)**		**API 50CH (carbohydrate degradation)**			
Alkaline phosphatase	-	β-galactosidase	+	Nitrate reduction	+	Glycerol	-	Salicin	-
Esterase (C4)	+	L-arginine	-	Indole production	-	Erythritol	-	D-cellobiose	-
Esterase Lipase (C8)	-	L-lysine	-	Glucose fermentation	-	D-arabinose	-	D-maltose	-
Lipase (C14)	-	L-omithine	-	L-arginine	-	L-arabinose	+	D-lactose	-
Leucine arylamidase	+	Trisodium citrate	+	Urea	+	D-ribose	-	D-melibiose	-
Valine arylamidase	-	Sodium thiosulfate	-	Esculin ferric citrate	+	D-xylose	-	D-saccharose (sucrose)	-
Cystine arylamidase	-	Urea	+	Gelatin hydrolysis	-	L-xylose	-	D-trehalose	-
Trypsin	-	L-tryptophan utilization	-	*p*-nitrophenyl-β-D-galactopyranosidase	+	D-adonitol	-	Inulin	-
α-chymotrypsin	-	L-tryptophan production	-	D-glucose	-	Methyl-β- D-Xylopyranoside	-	D-melezitose	-
Acid phosphatase	+	Sodium pyruvate	+	L-arabinose	-	D-galactose	-	D-raffinose	-
Naphthol-AS-BI-phosphohydrolase	-	Gelatin	-	D-mannose	-	D-glucose	+	Amidon (starch)	-
α-galactosidase	-	D-glucose	+	D-mannitol	-	D-fructose	-	Glycogen	-
β-galactosidase	-	D-mannitol	-	N-acetyl-glucosamine	-	D-mannose	-	Xylitol	-
β-glucuronidase	-	Inositol	-	D-maltose	-	L-sorbose	-	Gentiobiose	-
α-glucosidase	+	D-sorbitol	-	Potassium gluconate	-	L-rhamnose	-	D-Turanose	-
β-glucosidase	+	L-rhamnose	-	Capric acid	-	Dulcitol	-	D-Lyxose	-
N-acetyl-β-glucosaminidase	-	D-saccharose	-	Adipic acid	-	Inositol	-	D-Tagatose	-
α-mannosidase	-	D-melibiose	-	Malic acid	-	D-mannitol	-	D-Fucose	+
α-fucosidase	-	D-saccharose	-	Trisodium citrate	-	D-sorbitol	-	L-fucose	-
		D-melibiose	-	Phenylacetic acid	-	Methyl-α- D-mannopyranoside	-	D-arabitol	-
		Amygladine	-	Oxidase	-	Methyl-α-D-glucopyranoside	-	L-arabitol	-
		L-arabinose	+			N-acetylglucosamine	-	Potassium gluconate	-
						Amygdalin	-	Potassium 2-ketogluconate	-
						Arbutin	+	Potassium 5-ketogluconate	-
						Esculin ferric citrate	+		
