# Supplementary material for: Genome Analysis of Celeribacter sp. PS-C1 Isolated from Sekinchan Beach in Selangor, Malaysia, Reveals Its β-Glucosidase and Licheninase Activities
Source: Microorganisms. 2022 Feb 10;10(2):410. doi: 10.3390/microorganisms10020410 (PMC8874975; doi:10.3390/microorganisms10020410)
Supplement: Supplementary file 1 [file microorganisms-10-00410-s001.zip › Table S5.pdf]

Table S5. List of NCBI GenBank/RefSeq accession numbers for glycoside hydrolases (GHs) in *Celeribacter* genomes.

Category (CAZy GH family)	Predicted function (EC number)	Identity within *Celeribacter* genomes (%)														
		**PS-C1**	**P73^T**	**TSPH2**	**IMCC12053^T**	**LH4**	**DSM27375^T**	**DSM26471^T**	**DSM100434^T**	**NH195^T**	**ZXM137^T**	**G3M19**	**ASW11-22**	**EaN35-2**	**B30**	**HF31**
**Cellulose-degrading enzyme** (GH1)	β-glucosidase (3.2.1.21)	WP_219784598.1	WP_043869591.1	WP_096805185.1	WP_062215664.1	CP028475.1_711	WP_074640371.1	WP_090058677.1	WP_107816655.1	LRUC01000013.1_57	LRUD01000054.1_137	JAHKPE010000013.1_151	WP_224504147.1	JAIVLK010000002.1_304	AMRK01000003.1_188	WP_167600635.1
**Β-glucan-degrading enzyme** (GH8)	Endo-1,3(4)-β-glucanase (3.2.1.6)	-	-	-	-	-	-	-	-	-	-	-	-	-	-	WP_167603039.1
(GH16)	Llicheninase (3.2.1.73)	WP_219783980.1	WP_052453328.1	-	-	-	-	-	-	-	-	JAHKPE010000013.1_376	-	JAIVLK010000001.1_588	-	WP_217703106.1
(GH26)	Endo-β-1,3-1,4-glucanase (3.2.1.73)	-	-	WP_096805185.1	-	CP028476.1_48	-	WP_143093075.1	WP_107816665.1	LRUC01000013.1_47	-	-	-	-	-	-

PS-C1: Strain PS-C1; EaN35-2: *Celeribacter naphthalenivorans* EaN35-2; HF31: *Celeribacter* sp. HF31; NH195^T: *Celeribacter ethanolicus* NH195^T [27]; TSPH2: *Celeribacter ethanolicus* TSPH2 [30]; ZXM137^T: *Celeribacter halophilus* ZXM137^T; G3M19: *Celeribacter halophilus* G3M19; DSM100434^T: *Celeribacter persicus* DSM100434^T [26]; DSM26471^T: *Celeribacter neptunius* DSM26471^T [19]; ASW11-22: *Celeribacter* sp. ASW11-22; P73^T: *Celeribacter indicus* P73^T [16,24]; B30: *Celeribacter baekdonensis* B30; DSM27375^T: *Celeribacter baekdonensis* DSM27375^T; LH4: *Celeribacter baekdonensis* LH4 [32]; IMCC12053^T: *Celeribacter marinus* IMCC12053^T [25,31]. Characteristics scored as: -, absence. ^{T} type strain.

Table S5. *Continued.*

Category (CAZy GH family)	Predicted function (EC number)	Identity within *Celeribacter* genomes (%)														
		**PS-C1**	**P73^{T}**	**TSPH2**	**IMCC12053^{T}**	**LH4**	**DSM27375^{T}**	**DSM26471^{T}**	**DSM100434^{T}**	**NH195^{T}**	**ZXM137^{T}**	**G3M19**	**ASW11-22**	**EaN35-2**	**B30**	**HF31**
**Hemicellulose-degrading enzyme**																
(GH2)	β-mannosidase (3.2.1.25)	-	-	WP_096805090.1	-	CP028475.1_1194	WP_074645874.1	-	WP_107815347.1	LRUC01000045.1_116	-	-	-	-	AMRK01000004.1_168	-
(GH26)	β-1,3-xylanase (3.2.1.32)	WP_219785562.1	-	-	-	-	-	-	-	-	-	-	-	-	-	WP_167600267.1
(GH36)	α-galactosidase (3.2.1.22)	-	-	-	-	-	-	-	-	-	LRUD01000026.1_120	JAHKPE010000030.1_96	-	-	-	-
(GH43)	β-xylosidase (3.2.1.37)	-	-	-	WP_062214939.1	-	-	-	-	-	-	-	-	-	-	-
(GH51)	α-L-arabinofuranosidase (3.2.1.55)	-	-	-	-	-	-	-	-	-	-	-	-	JAIVLK010000001.1_588	-	-

PS-C1: Strain PS-C1; EaN35-2: *Celeribacter naphthalenivorans* EaN35-2; HF31: *Celeribacter* sp. HF31; NH195^{T}: *Celeribacter ethanolicus* NH195^{T} [27]; TSPH2: *Celeribacter ethanolicus* TSPH2 [30]; ZXM137^{T}: *Celeribacter halophilus* ZXM137^{T}; G3M19: *Celeribacter halophilus* G3M19; DSM100434^{T}: *Celeribacter persicus* DSM100434^{T} [26]; DSM26471^{T}: *Celeribacter neptunius* DSM26471^{T} [19]; ASW11-22: *Celeribacter* sp. ASW11-22; P73^{T}: *Celeribacter indicus* P73^{T} [16,24]; B30: *Celeribacter baekdonensis* B30; DSM27375^{T}: *Celeribacter baekdonensis* DSM27375^{T}; LH4: *Celeribacter baekdonensis* LH4 [32]; IMCC12053^{T}: *Celeribacter marinus* IMCC12053^{T} [25,31]. Characteristics scored as: -, absence. ^{T} type strain.

Table S5. *Continued.*

Category (CAZy GH family)	Predicted function (EC number)	Identity within *Celeribacter* genomes (%)														
		**PS-C1**	**P73^T**	**TSPH2**	**IMCC12053^T**	**LH4**	**DSM27375^T**	**DSM26471^T**	**DSM100434^T**	**NH195^T**	**ZXM137^T**	**G3M19**	**ASW11-22**	**EaN35-2**	**B30**	**HF31**
**Starch-degrading enzyme**																
(GH13)	α-amylase (3.2.1.1)	-	-	-	-	-	-	-	-	-	LRUD01000012.1_184	JAHKPE010000031.1_875	-	-	AMRK01000009.1_1	-
(GH13)	Oligo-1,6-glucosidase (3.2.1.10)	-	-	-	-	-	-	-	-	-	-	-	-	-	AMRK01000009.1_2	-
(GH13)	α-glucosidase (3.2.1.20)	WP_219784593.1	WP_043871237.1	WP_096805181.1	WP_062215660.1	CP028475.1_717	WP_143026706.1	WP_090058665.1	WP_107816651.1	LRUC01000045.1_116	LRUD01000054.1_143	JAHKPE010000013.1_156	WP_224504152.1	JAIVLK010000002.1_309	AMRK01000003.1_183	WP_167600630.1

PS-C1: Strain PS-C1; EaN35-2: *Celeribacter naphthalenivorans* EaN35-2; HF31: *Celeribacter* sp. HF31; NH195^T: *Celeribacter ethanolicus* NH195^T [27]; TSPH2: *Celeribacter ethanolicus* TSPH2 [30]; ZXM137^T: *Celeribacter halophilus* ZXM137^T; G3M19: *Celeribacter halophilus* G3M19; DSM100434^T: *Celeribacter persicus* DSM100434^T [26]; DSM26471^T: *Celeribacter neptunius* DSM26471^T [19]; ASW11-22: *Celeribacter* sp. ASW11-22; P73^T: *Celeribacter indicus* P73^T [16,24]; B30: *Celeribacter baekdonensis* B30; DSM27375^T: *Celeribacter baekdonensis* DSM27375^T; LH4: *Celeribacter baekdonensis* LH4 [32]; IMCC12053^T: *Celeribacter marinus* IMCC12053^T [25,31]. Characteristics scored as: -, absence. ^{T} type strain.
