# Supplementary figures and images for: Genome Analysis of Celeribacter sp. PS-C1 Isolated from Sekinchan Beach in Selangor, Malaysia, Reveals Its β-Glucosidase and Licheninase Activities
Source: Microorganisms. 2022 Feb 10;10(2):410. doi: 10.3390/microorganisms10020410 (PMC8874975; doi:10.3390/microorganisms10020410)

# STARCH AND SUCROSE METABOLISM

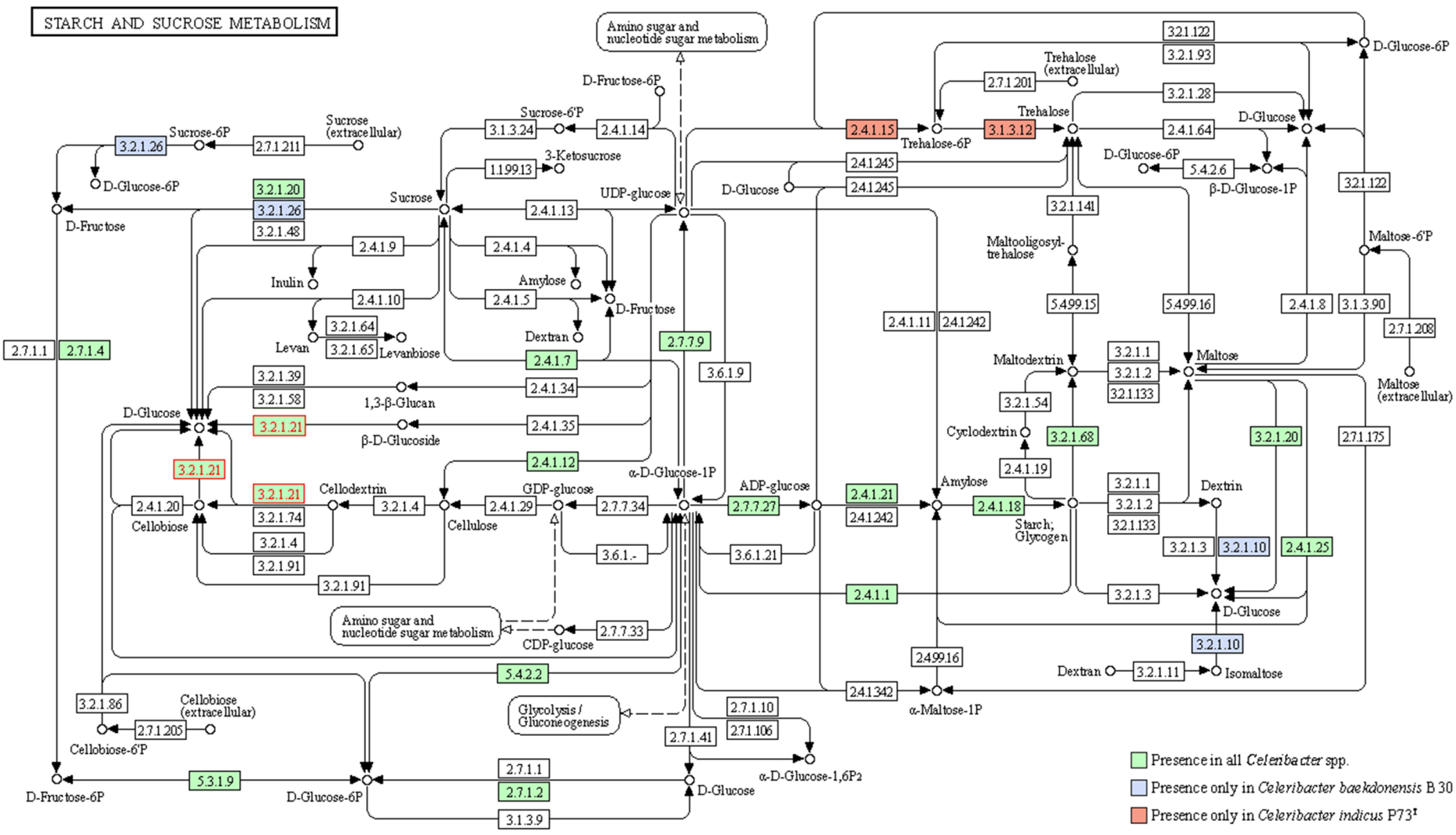

Supplement: Supplementary file 1 [file microorganisms-10-00410-s001.zip › Figure S1.pdf]
